# Community structure and metabolite profiles of psychrophiles and thermophiles in bovine raw milk from arid inland areas

**DOI:** 10.1128/aem.01065-25

**Published:** 2026-05-15

**Authors:** Tingting Huang, Ning Wang, Xia Chen, Xiao Wang, Ruiqi Wang, Meiling Jin, Danyang Huang, Yanan Qin, Junqiang Wu, Lulu Wang, Hussain Akbar, Haitao Yue

**Affiliations:** 1 College of Life Science and Technology, Xinjiang University 47907, Urumqi, China; 2 School of Intelligence Science and Technology, Xinjiang University 47907, Urumqi, China; 3 College of Pharmacy, Xinjiang University 47907, Urumqi, China; Anses, Maisons-Alfort Laboratory for Food Safety, Maisons-Alfort, France

**Keywords:** raw cow’s milk, psychrophiles, thermophiles, metabolites

## Abstract

This study provides a regional assessment of bacterial communities and associated metabolite profiles in raw cow’s milk collected from seven commercial farms across southern and northern Xinjiang, an arid inland milk-producing region of China. By comparatively analyzing psychrophilic and thermophilic bacterial assemblages, this work aimed to clarify their community composition, explore their potential environmental origins, and identify metabolites that differ between these two microbial groups with possible implications for milk quality and safety. To achieve this, we combined 16S rRNA gene sequencing, culture-based isolation of representative strains, and untargeted liquid chromatography–mass spectrometry metabolomics. At the phylum level, three dominant bacterial phyla were identified in samples from southern farms, while four dominant phyla were observed in northern farms; at the genus level, Pseudomonas and Acinetobacter were among the most abundant taxa associated with psychrophilic or thermophilic conditions. Differential metabolomic analysis revealed 59 metabolites that were consistently upregulated in samples associated with thermophilic communities and 2 that were downregulated, whereas samples associated with psychrophilic communities showed 1consistently downregulated metabolite. One metabolite feature was annotated as 3,4-methylenedioxyamphetamine based on database matching; given the inherent uncertainty of untargeted annotations, this identification requires further targeted validation before definitive safety conclusions can be drawn. Pathway enrichment analysis implicated arginine biosynthesis and galactose metabolism, which may be related to enzymatic activities involved in milk spoilage. Overall, this study offers a regionally focused characterization of psychrophilic and thermophilic microbial and metabolite patterns in raw milk from arid inland Xinjiang and identifies microbial and metabolic features that warrant further targeted investigation for dairy quality control.

**IMPORTANCE:**

Raw milk from arid inland regions represents a unique ecological niche in which microbial survival, metabolic activity, and contamination dynamics differ from those in humid or temperate dairy systems. By jointly characterizing psychrophilic and thermophilic microbial communities and their associated metabolite profiles, this study provides an integrated microbe–metabolite perspective on raw milk quality under arid production conditions. The coupling of microbial source tracking with metabolomic analysis offers a framework for linking contamination sources to functional and chemical consequences, extending beyond traditional taxonomic surveys. The detection of metabolites with potential toxicological relevance highlights the importance of integrating chemical risk screening into routine microbiological assessments, while also underscoring the need for targeted confirmation in food safety surveillance. More broadly, this work demonstrates how combined microbial–metabolite approaches can support evidence-based hygiene management and risk prevention strategies in dairy production systems, with particular relevance for arid and semi-arid agricultural regions.

## INTRODUCTION

**Fig 1:**
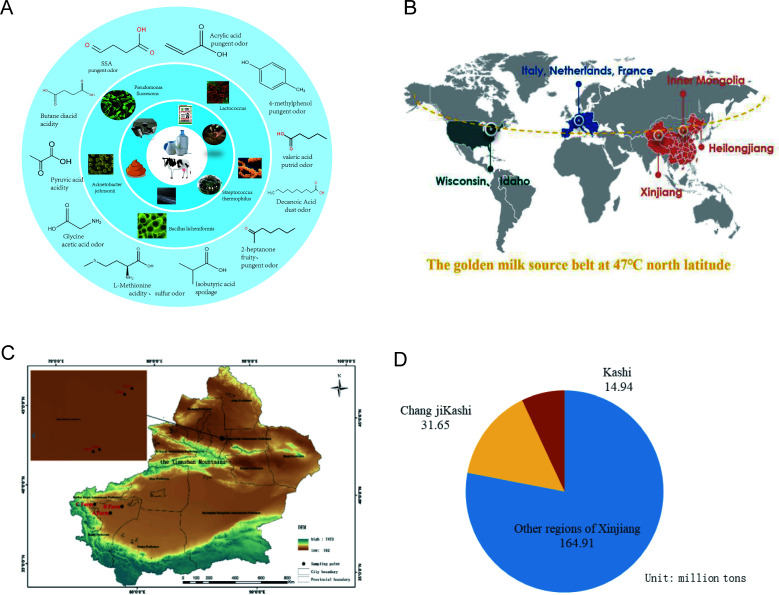
Distribution of raw cow’s milk collection points in Xinjiang and a summary of the research content. (**A**) Sources of bacterial contamination in milk and its associated metabolites. (**B**) The global golden milk source region. The map was created using ArcGIS (version 10.8) software, with base map from MapCruzin. (**C**) Map of raw cow milk sample collection sites. The map was created using ArcGIS (version 10.8) software, with base map from National Geomatics Center of China. (**D**) Milk production in the Kashgar and Changji regions of Xinjiang in 2021 (source: 2022 China Statistical Yearbook; 2021 Statistical Bulletin of the National Economic and Social Development of Changji Hui Autonomous Prefecture; 2021 Statistical Bulletin of the National Economic and Social Development of Kashgar Region).

Milk is a vital global dietary staple and serves as a foundational ingredient in dairy production (1). As of 2018, the global per capita daily consumption of dairy products averaged 88 grams (2). China’s agricultural and food processing industries have significantly enhanced the production of milk and dairy products. According to the 2021 National Economic and Social Development Statistics Bulletin from the Xinjiang Uygur Autonomous Region, China emerged as the third largest milk producer in the world in 2020, with a production volume of 36.83 million tons. As a key milk-producing region in China, the dairy industry has become one of the essential economic pillars for the development of Xinjiang. Between 2011 and 2021, milk production exhibited a steady increase, reaching 2.115 million tons in 2021, which accounted for 5.7% of the national production (Fig. 1A and B).

The nutrient-rich composition of milk not only provides essential benefits but also creates an environment conducive to microbial proliferation (3). The quality of raw cow’s milk is a critical factor that affects the entire dairy processing chain and directly impacts the quality and safety of milk and its derived products (4). Research has demonstrated that seasonal variations and geographic origin significantly contribute to differences in the microbiota of raw milk (5). The presence of bacteria in raw cow’s milk from healthy cows primarily results from contamination from the external environment; thus, microbial contamination of milk can vary based on the geography of the pasture (6). Additionally, it can be influenced by milking practices (7) and herd management strategies (8).

Breeding conditions and regional factors significantly contribute to the diversity of bacterial community composition in raw cow’s milk, as well as influence the distribution of psychrophiles and thermophiles within it (6, 9, 10). The activity and impact of thermophiles in contaminated milk and dairy products have been limited by the widespread use of low-temperature storage, transportation systems, and autoclaving techniques in the dairy industry. However, these conditions also promote the prevalence of psychrophiles (11), which further affects the quality of dairy products (12). Biofilms are matrices composed of extracellular polymeric substances (EPS) that provide protection to cells (13). This biofilm protection renders cells resistant to cleaning and sanitation efforts, allowing harmful microorganisms in food to potentially cause food poisoning. Low temperatures favor the production of extracellular polymeric substances, thereby increasing biofilm formation. Bacteria adhere to various surfaces through biofilms, leading to equipment contamination, increased costs, spoilage of dairy products, and even foodborne diseases (14, 15). Additionally, some thermophiles, such as *Bacillus cereus* spores, can survive heat sterilization and subsequently multiply in the finished product, potentially compromising the quality of dairy products (16).

Xinjiang, located in western China, encompasses a vast arid region characterized by agricultural practices typical of the world’s arid zones (17, 18). The rapidly growing dairy industry and the expansion of livestock farms in Xinjiang have resulted in fluctuations in the quality of raw milk during production and processing. For instance, post-processing issues such as sack swelling and hydrolytic rancidity can occur, with psychrophiles and thermophiles being significant contributors to these problems. Addressing bacterial contamination requires an investigation into the bacterial community structure and origins within milk, as well as an assessment of how psychrophiles and thermophiles impact the quality of raw milk and its subsequent dairy products. Although the bacterial communities and types of psychrophiles and thermophiles in milk and dairy products have been studied, variations in environmental bacterial structures indicate that agricultural and production settings can introduce distinct microorganisms into milk. To date, a comprehensive analysis of psychrophiles and thermophiles in Xinjiang’s raw milk has not been conducted.

This study investigated psychrophiles and thermophiles in raw milk from Xinjiang, analyzed their potential hazards, and aimed to mitigate milk contamination. We selected seven representative farms in the Kashgar region (southern Xinjiang) and the Changji region (northern Xinjiang) to collect raw cow’s milk, which accounted for 7% and 15% of the annual milk production in Xinjiang, respectively. Farms A and B were free-range herding operations, while farms C, D, E, and F were intensive farming operations (Fig. 1C and D). We employed a combination of high-throughput sequencing and traditional culture methods to analyze the psychrophiles and thermophiles. Additionally, environmental samples collected from farms A, B, and C in southern Xinjiang were selected to trace external contamination by bacteria. Subsequently, we conducted a metabolite analysis of the identified psychrophiles and thermophiles in the raw milk.

## MATERIALS AND METHODS

### Raw milk sample collection

A total of 21 raw milk samples (100 mL each) were collected from 3 livestock farms (A, B, and C) located in Yebu Lake, Bachu, and Shushi counties of the Kashgar region, as well as from four livestock farms (D, E, F, and G) in Hutubi County within the Changji Hui Autonomous Prefecture. The samples were collected in July 2021, then frozen, and transported to the laboratory, where they were stored at −80°C until DNA extraction and metabolomics analysis.

Milk sampling was conducted during 2023–2024, with major restrictions occurring in 2023 due to COVID-19 control measures. The study covered geographically distant production regions in southern Xinjiang (Kashgar) and northern Xinjiang (Changji). In Kashgar, samples were collected from three large Nanda farms and one Xiyuchun farm; in Changji, samples were collected from three Xiyuchun farms. All sampled farms are large-scale, standardized operations, and the farms selected for sampling adhere to national sanitary and technical specifications for dairy farms (GB 16568-2006 and related Ministry of Agriculture technical guidelines). Because external access to production zones was limited in 2023, sampling was principally performed by authorized and trained farm personnel following unified standard operating procedures (aseptic collection, cold-chain preservation, and timely transport to the laboratory). Three biological replicates were obtained from each farm.

### Collection of environmental samples for livestock farms

Samples of forage, barn bedding, feces, and swabs from milking machines were collected from three livestock farms (A, B, and C) in Kashgar. These farms employ different farming methods: farms A and B operate as free-range herding operations, while farm C functions as an intensive farming facility. The variations in farming practices result in differences in livestock activities, foraging methods, and environmental conditions, which subsequently influence the microbiological contamination of milk. However, the shared geographical location of these farms mitigates the impact of regional factors. Three replicates of each sample were collected and placed in sterile bags and then transported to the laboratory under refrigeration for DNA extraction. The extracted DNA was subsequently sent to Majorbio Bio-Pharm Technology Co., Ltd. (Shanghai, China) for sequencing.

### Data processing of bacterial diversity results in milk

DNA was extracted from the samples using the FastDNA Spin Kit for Soil (USA) and sequenced on the MiSeq PE300/NovaSeq PE250 platform (Majorbio Bio-Pharm Technology Co., Ltd., Shanghai, China). The raw sequences underwent quality control (QC) using fastp (https://github.com/OpenGene/fastp, version 0.20.0), followed by splicing with FLASH (https://ccb.jhu.edu/software/FLASH/, version 1.2.7) (19). Operational taxonomic unit (OTU) clustering was performed using UPARSE (http://drive5.com/uparse/, version 7.1) at a threshold of 97% similarity. Each sequence was annotated for species classification using the RDP classifier (https://sourceforge.net/projects/rdp-classifier/, version 2.2) and compared to the Silva 16S rRNA database (v138), with a comparison threshold set at 70%. Alpha diversity analysis was conducted using Mothur (v.1.30.2) to assess species abundance and diversity within the samples, while beta diversity analysis was performed using QIIME 1 (v.1.9.1) to compare the community composition of the samples. Additionally, the species composition of the samples was analyzed using R (version 3.3.1).

The FastDNA Spin Kit for Soil (MP Biomedicals, USA) was chosen after a systematic comparison of multiple commercial DNA extraction kits across different sample types routinely analyzed in our laboratory, including human feces, environmental soil, and raw milk. This comparative evaluation demonstrated that the kit provided the highest DNA yield and consistency for raw milk samples, while efficiently removing PCR inhibitors such as fats and proteins. To further optimize recovery, minor modifications were made to the lysis step, including extended bead-beating and an additional centrifugation step. The robustness of this method has also been demonstrated in our previous metagenomic work (20), supporting its suitability for complex microbial communities.

### Screening and identification of psychrophiles and thermophiles in raw milk

A total of 1 mL of raw milk was mixed in a test tube containing 9 mL of normal saline solution and then diluted to 10^−7^ using a series of gradient dilution methods. Subsequently, 100 μL of each diluted sample was plated on MPC agar medium (*n* = 3). Psychrophiles were cultured at 6.5 ± 0.5°C for 10 days, while thermophiles were cultured at 55 ± 1 °C for 48 ± 2 h (21, 22). The individual colonies exhibiting distinct colony morphologies were randomly selected, cultured, and purified twice on MPC medium. The purified strains were then cultured and preserved in a shake flask at 150 rpm in tryptic soy broth medium.

The genomic DNA of psychrophiles and thermophiles was extracted using a bacterial DNA extraction kit. The 16S rRNA gene of the preliminarily screened psychrophiles and thermophiles was amplified and sequenced using the universal primers 27F (5′-GAGTTTGATCCTGGCTCAG-3′) and 1492R (5′-GGTTACCTTGTTACGACTT-3′). The PCR conditions were as follows: an initial denaturation at 95°C for 3 min, followed by 40 cycles of denaturation at 95°C for 30 s, annealing at 56°C for 45 s, and extension at 72°C for 90 s, with a final extension at 72°C for 10 min. The PCR products were visualized using 1.2% agarose gel electrophoresis and sequenced by Majorbio Bio-Pharm Technology Co., Ltd. (Shanghai, China). The 16S sequencing results were aligned with the NCBI database using BLAST to identify these strains (23).

### Enzyme activity and milk spoilage potential of psychrophiles and thermophiles

To evaluate spoilage potential, protease and lipase activities of psychrophilic and thermophilic isolates were assessed after growth in sterilized milk. Strains with the highest enzyme activities were considered the most relevant to milk spoilage and were selected for subsequent re-inoculation experiments. Detailed procedures followed previously published protocols (24).

### Sterilized milk back inoculation of psychrophiles and thermophiles

Seven psychrophiles and seven thermophiles showing the strongest protease and lipase activities (see previous paragraph) were inoculated into sterilized milk at 2% concentration. Cultures were incubated under the same conditions described above and subsequently subjected to metabolite profiling.

### Raw cow’s milk metabolite extraction and LC–MS analysis

The experimental samples were thawed at 4°C and vortexed for 1 min to ensure homogeneity. An aliquot of each sample was mixed with 200 μL methanol and 200 μL methyl tert-butyl ether, vortexed, and centrifuged at 12,000 rpm for 10 min at 4°C. The supernatant was filtered through a 0.22 μm membrane and used for metabolite detection. A pooled quality control (QC) sample was prepared by combining 10 μL from each extract.

Metabolomic profiling was performed on a Thermo Scientific Q Exactive Orbitrap mass spectrometer coupled with an Ultimate 3000 UHPLC system (Thermo Fisher Scientific, USA). Chromatographic separation was achieved using a Waters ACQUITY UPLC HSS T3 column (100 mm × 2.1 mm, 1.8 μm). The mobile phases consisted of (A) water with 0.1% formic acid and (B) acetonitrile with 0.1% formic acid. The elution gradient was programmed as follows: 0–2 min, 2% B; 2–12 min, 2%–98% B; 12–15 min, 98% B; 15–15.1 min, 98%–2% B; and 15.1–18 min, 2% B for column re-equilibration. The flow rate was 0.3 mL/min, the injection volume was 5 μL, and the column temperature was maintained at 40°C.

Mass spectrometry was conducted in both positive and negative electrospray ionization modes, with a scan range of *m*/*z* 70–1,050. The capillary temperature was set to 320°C, spray voltage to 3.5 kV, sheath gas at 35 arb units, and auxiliary gas at 10 arb units.

Raw liquid chromatography–mass spectrometry (LC–MS) data were processed using Compound Discoverer software (Thermo Fisher Scientific) for peak detection, alignment, and normalization. Metabolites were annotated based on accurate mass, MS/MS fragmentation patterns, and comparison with the Human Metabolome Database (HMDB) and Kyoto Encyclopedia of Genes and Genomes (KEGG) database. Multivariate analyses, including principal component analysis (PCA) and partial least squares discriminant analysis, were performed to assess metabolic differences among groups.

### Statistical analysis

Alpha diversity indices (Chao and abundance-based coverage estimator [ACE]) of microbial communities were compared among farms using one-way analysis of variance (ANOVA), followed by Tukey’s *post hoc* test. Beta diversity differences were visualized by principal coordinate analysis based on Bray–Curtis distances and assessed using permutational multivariate analysis of variance. For metabolomic profiling, peak intensities were log-transformed prior to statistical testing. Differential metabolites between psychrophilic and thermophilic groups were identified using Student’s *t*-test or ANOVA, with false discovery rate correction applied to account for multiple comparisons. Results with corrected *P* (*q*-value) < 0.05 were considered statistically significant.

## RESULTS

### Microbial community composition of raw cow’s milk

To investigate the differences in microbial community structure of raw milk from arid regions and their potential impact on dairy products, we selected seven representative livestock farms in Kashgar, located in southern Xinjiang, and Changji, situated in northern Xinjiang, to collect raw milk for community analysis.

A total of 16 phyla, 23 classes, 55 orders, 102 families, 83 genera, 260 species, and 419 OTUs were annotated from 21 raw milk samples collected from livestock farms in southern and northern regions. The results of the alpha diversity analysis are presented in Table 1. The Chao and ACE indices for raw milk samples from C livestock farms in the southern border region were lower than those of the other two livestock farms, indicating a reduced abundance of microbial flora in C livestock farms. In contrast, samples from E and F livestock farms in northern Xinjiang exhibited relatively high Chao and ACE indices, suggesting a greater abundance of microbial flora. Furthermore, the smallest Simpson index and the largest Shannon index indicated the highest microbial diversity in the raw milk from E livestock farms. Additionally, the ACE, Chao, and Shannon indices for southern Xinjiang livestock farms were generally lower compared to those of northern Xinjiang livestock farms, while the Simpson index was higher, suggesting lower richness and diversity in the raw milk from southern livestock farms.

**TABLE 1:** Microbial alpha diversity indices in cow’s milk from livestock farms

Livestock farm	ACE	Chao	Shannon	Simpson
A	154.34 ± 18.1	151.94 ± 19.01	1.5 ± 0.38	0.37 ± 0.06
B	131.6 ± 15.53	124.08 ± 4.64	1.08 ± 0.06	0.44 ± 0.01
C	103.31 ± 22.01	97.42 ± 19.72	2.2 ± 0.05	0.19 ± 0.01
D	256.92 ± 24.67	266.18 ± 12.98	2.99 ± 0.25	0.1 ± 0.02
E	305.79 ± 15.07	315.5 ± 16.81	4.01 ± 0.14	0.04 ± 0
F	275.99 ± 44.51	269.5 ± 47.59	2.37 ± 0.37	0.18 ± 0.05
G	264.51 ± 1.02	259.69 ± 0.31	2.22 ± 0.05	0.24 ± 0.02

**Fig 2:**
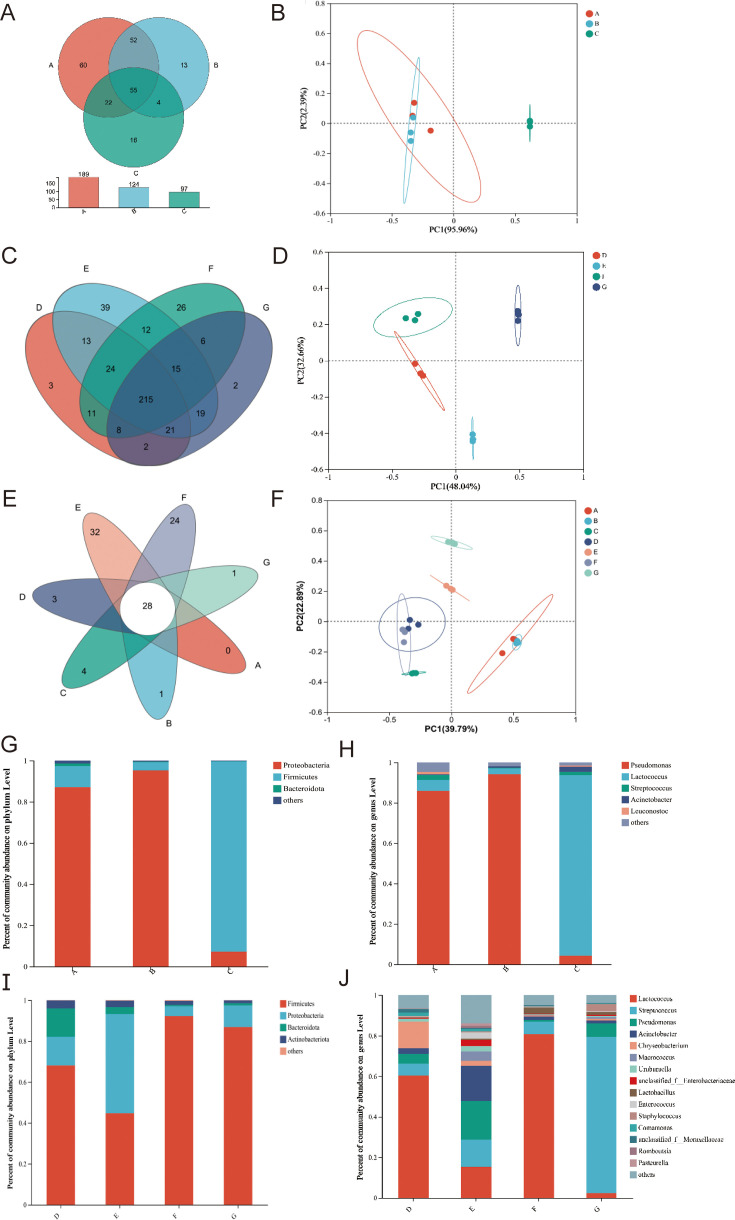
Analysis of bacterial community structure in raw cow’s milk. (**A–B**) OTU Venn diagrams illustrating the bacterial communities in raw cow’s milk from three livestock farms in southern Xinjiang, along with principal coordinate analysis (PCoA) of the bacterial community structure in these livestock farms. (**C–D**) OTU Venn diagrams depicting the bacterial communities in raw cow’s milk from four livestock farms in northern Xinjiang, accompanied by PCoA of the bacterial community structure in these livestock farms. (**E–F**) OTU Venn diagrams representing seven livestock farms and PCoA of the bacterial community structure across these seven livestock farms. (**G–J**) Structural composition at the phyla and genus levels, along with the relative abundance of bacterial communities in livestock farms from southern and northern Xinjiang.

As illustrated in Fig. 2A through F, the OTU numbers of the samples from the south were significantly smaller than those from the north. The total number of OTUs in the 3 samples from the southern livestock farms was 55, compared to 215 in the northern samples. Additionally, the total number of OTUs shared among the 7 farms was 28. We also observed that the raw milk from nearby livestock farms exhibited a similar bacterial community structure; for instance, the microbial flora of farms A and B (located in southern Xinjiang) was analogous, yet significantly different from the samples collected from the other five farms. This finding underscores the shaping effect of the geographical environment.

Three predominantly dominant phyla were identified in raw cow’s milk from southern livestock farms: Proteobacteria, Firmicutes, and Bacteroidetes. Specifically, in livestock farm A, the bacterial phyla were predominantly Proteobacteria at 87.10%, followed by Firmicutes at 10.23% and Bacteroidetes at 1.25%. In comparison, livestock farm B contained 95.38% Proteobacteria, 3.80% Firmicutes, and 0.32% Bacteroidetes, while livestock farm C comprised 92.36% Proteobacteria, 7.24% Firmicutes, and only 0.10% Bacteroidetes (Fig. 2G).

*Pseudomonas*, *Lactococcus*, *Streptococcus*, *Acinetobacter*, and *Leuconostoc* were identified as the dominant genera. In livestock farm A, the five most abundant genera were *Pseudomonas* at 85.94%, *Lactococcus* at 5.40%, *Streptococcus* at 2.43%, *Leuconostoc* at 1.20%, and *Acinetobacter* at 0.33%. In livestock farm B, the leading genus included *Pseudomonas*, which dominated at 94.15%, followed by *Lactococcus* at 2.88%, *Streptococcus* at 0.33%, and *Acinetobacter* at 0.77%. For livestock farm C, *Lactococcus* was the most abundant genus at 89.36%, followed by *Pseudomonas* at 4.29%, *Streptococcus* at 1.68%, *Acinetobacter* at 2.56%, and *Leuconostoc* at 0.57% (Fig. 2H).

While four dominant phyla were identified in the Northern Rangelands, including Proteobacteria, Firmicutes, Bacteroidetes, and Actinobacteria, the specific distributions were as follows: the samples from the D livestock farm contained 68.36% Firmicutes, 13.88% Proteobacteria, 13.74% Bacteroidetes, and 3.95% Actinobacteria. In contrast, the E livestock farm comprised 44.55% Firmicutes, 48.79% Proteobacteria, 3.28% Bacteroidetes, and 3.19% Actinobacteria. Notably, the abundance of Firmicutes was significantly higher in the farms F and G, reaching ratios of 92.05% and 86.90%, respectively (Fig. 2I).

At the genus level, the dominant taxa identified were *Lactococcus*, *Streptococcus*, *Pseudomonas*, *Acinetobacter*, *Chryseobacterium*, *Uruburuella*, *unclassified Enterobacteriaceae*, *Lactobacillus*, *Staphylococcus*, *Enterococcus*, *Comamonas*, *unclassified Moraxella*, *Romboutsia*, and *Pasteurell*a, collectively accounting for over 99% of the total. The five most abundant genera in livestock farm D were *Lactococcus* (60.62%), *Chryseobacterium* (13.19%), *Streptococcus* (5.82%), *Pseudomonas* (4.84%), and *Acinetobacter* (2.69%), which were consistent with the predominant genus observed in livestock farm F. In contrast, livestock farm E exhibited a different composition, with *Pseudomonas* (19.16%), *Lactococcu*s (17.38%), *Streptococcus* (15.31%), *Acinetobacter* (13.35%), and *Macrococcus* (4.75%) as the leading genera. Livestock farm G, however, was dominated by *Streptococcus* (77.10%), followed by *Pseudomonas* (6.76%), *Staphylococcus* (3.63%), *Lactococcus* (2.29%), and *Lactobacillus* (1.36%) (Fig. 2J).

### Traceability of bacteria in raw cow’s milk from livestock farms

**Fig 3:**
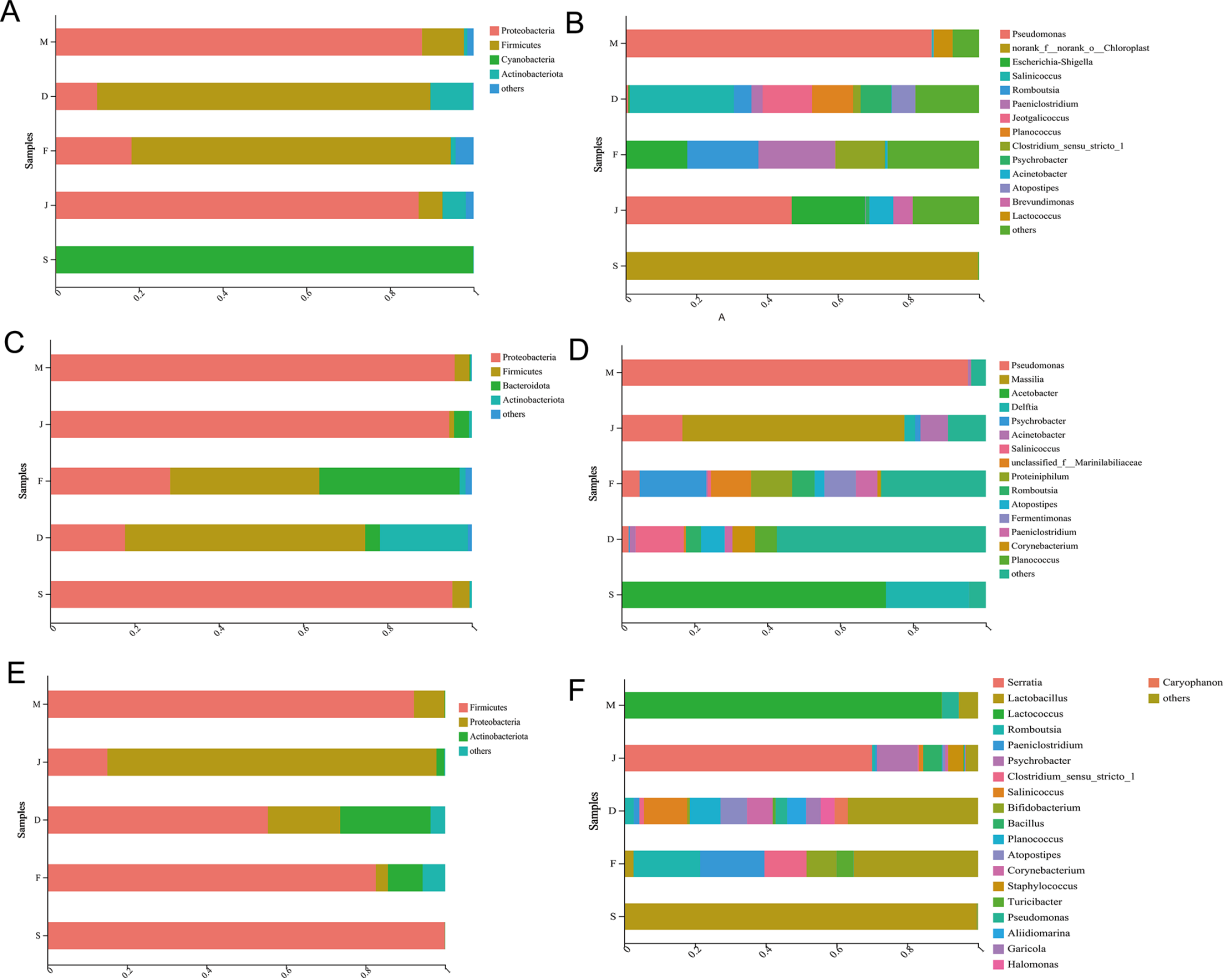
Composition and relative abundance of bacterial communities in raw cow’s milk and environmental samples. (**A and B**) Phylum-level (**A**) and genus-level (**B**) structural composition and relative abundances of bacterial communities in raw cow’s milk and environmental samples from livestock farm A. (**C and D**) Genus-level structural composition and relative abundances of bacterial communities in raw cow’s milk and environmental samples from livestock farm B. (**E and F**) Genus-level structural composition and relative abundances of bacterial communities in raw cow’s milk and environmental samples from livestock farm C. Bacterial communities were analyzed in raw cow’s milk (M), bedding (D), feces (F), milking machines (J), and forage (S) collected from southern farms.

To investigate the traceability of bacteria present in raw cow’s milk, we analyzed the proportions of dominant species in environmental samples at both the phylum and genus levels, which included bedding (D), feces (F), milking machines (J), and forage (S) samples. Proteobacteria constituted the largest proportion at both the phylum and genus levels in raw cow’s milk, with the primary source identified as the milking machine, followed by bedding (10%) and feces (18.2%). Firmicutes exhibited high proportions in bedding (79.7%) and feces (76.2%), while it accounted for only 5.7% in the milking machine. Bacteroidetes comprised 10.2%, 1.1%, and 5.6% of the microbial community in bedding, feces, and the milking machine, respectively (Fig. 3A). *Pseudomonas*, the main bacterium in raw cow’s milk from farm A, was found in the highest proportion in milking machine samples at 47% and was also present in trace amounts in bedding and feces at 0.73% and 0.02%, respectively. Conversely, *Lactococcus* was detected only in trace amounts in the bedding at 0.02% (Fig. 3B).

Proteobacteria, the most abundant phylum in raw cow’s milk from farm B, constituted the highest proportion of microorganisms in the milking machine and forage, accounting for 94.6% and 95.4%, respectively. This phylum was also detected in feces (28.4%) and bedding (28.7%). Firmicutes were predominantly found in bedding (57%) and feces (35.4%), with smaller quantities present in the milking machine (1.1%) and forage (4.1%). Bacteroidetes primarily originated from feces, comprising 33.3% of its microbial community, while bedding and the milking machine contributed additional sources, with Bacteroidetes percentages of 3.5% and 3.6%, respectively. Actinobacteria were mainly present in bedding (20.9%) and were also found in trace amounts in feces (1.4%), the milking machine (0.7%), and forage (0.6%) (Fig. 3C). Genus level analysis revealed similar results to those observed in livestock farm A, where the primary source of *Pseudomonas* was the milking machine, albeit at a significantly lower percentage (16.7%). *Pseudomonas* was also detected in feces (4.9%) and bedding (1.9%). *Acinetobacter* was present in 7.53%, 1.64%, and 0.40% of the milking machine, bedding, and feces, respectively (Fig. 3D).

In comparison, Firmicutes were the dominant phylum in raw milk from farm C, primarily originating from forage and fecal matter, which accounted for 99.9% and 82.6% of their microbial populations, respectively. Additionally, bedding and the milking machine may also serve as potential sources of Firmicutes, contributing 55.4% and 15.1% to their microbial community composition. The milking machine and bedding significantly influenced the presence of Aspergillus and Actinobacteria, which comprised 82.7% and 22.7% of their respective bacterial communities (Fig. 3E). *Lactobacillus* was the predominant microorganism in raw cow’s milk from farm C, followed by *Pseudomonas* and *Fusobacterium*. However, *Lactobacillus* was absent in all four environments, while *Pseudomonas* was primarily found in bedding at 3.3% and in feces at 0.1%. *Fusobacterium* was detected in feces and bedding at 1.7% and 1.6%, respectively (Fig. 3F).

**Fig 4:**
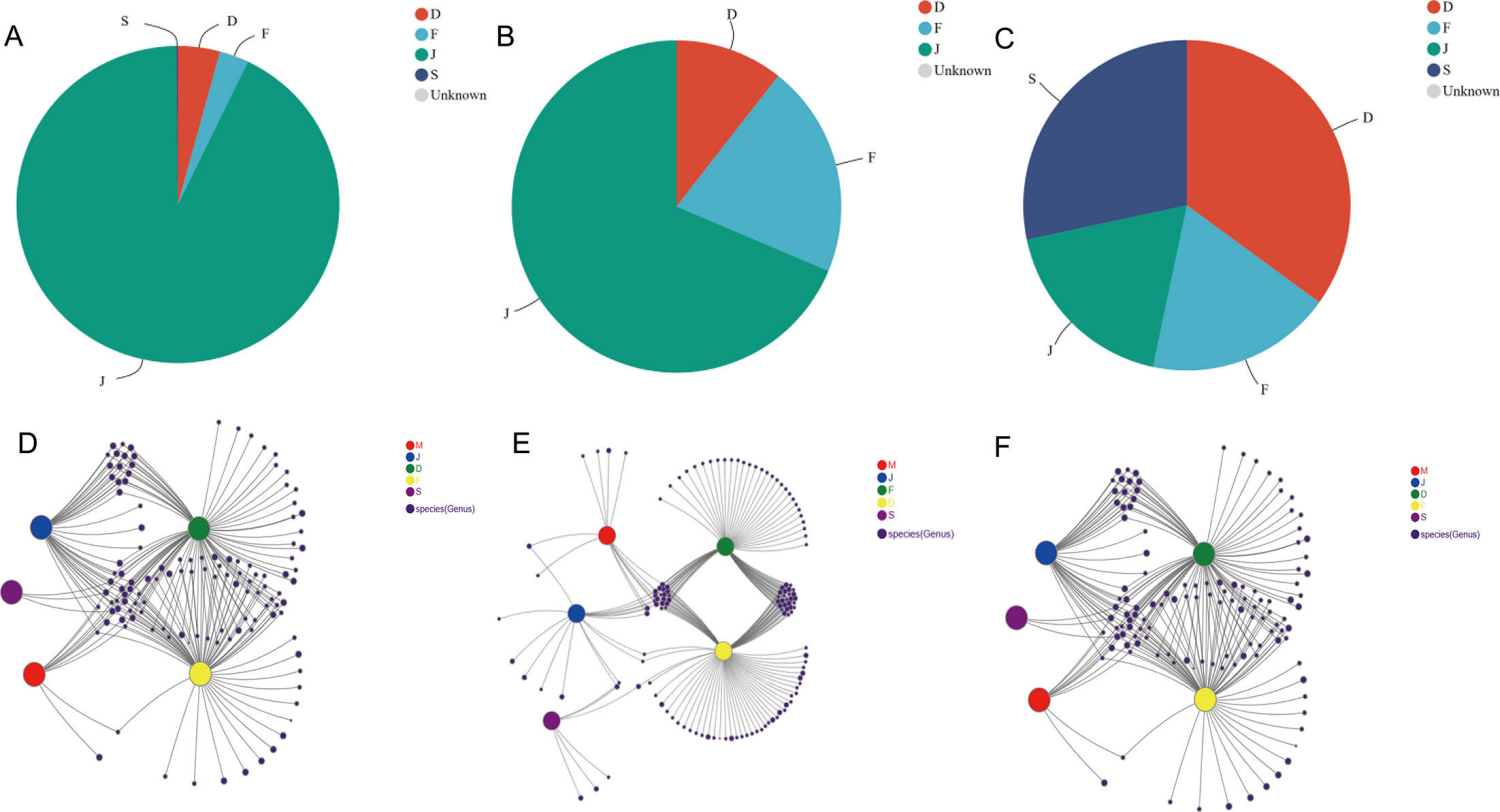
Traceability of bacterial communities in raw cow’s milk and the environment. (**A, B, C**) Percentage of microorganisms from environmental samples in raw cow’s milk from farms A, B, and C. (**D, E, F**) Co-occurrence networks diagrams of raw cow’s milk from farms A, B, and C, including bacteria from environmental samples.

Further traceability analysis of on-farm raw cow’s milk samples and environmental samples was conducted. The milking machines from livestock farm A exhibited a highly similar microbial community to that of the raw cow’s milk, which was significantly different from the other environmental samples. It contributed the highest percentage (1.18%) to the milk microbial community composition, followed by feces (0.08%), bedding (0.05%), and forage (0.01%). Fifteen genera were identified in both raw milk and the milk collection equipment, with notable examples including *Pseudomonas*, *Microbacterium*, *Flavobacterium aureum*, *Bacillus immobilis*, *Rhonnenbergia*, and *Bacillus chillingophilus*. Additionally, bedding was identified as the source of sixteen genera, including *Pseudomonas*, *Streptococcus*, *Microbacterium*, *Fusobacterium*, *Lactococcus*, *Turicibacter*, and *Rhombus*. Furthermore, 10 genera were traced back to fecal matter, while 2 were associated with forage. The primary genus identified in feces included *Fusobacterium*, *Enterococcus*, *Turicibacter*, and *Rhombus*, whereas *Microbacterium* and *Lactococcus* were predominantly found in forage (Fig. 4A and D).

In comparison, the microbial community of the raw cow’s milk from livestock farm B exhibited a coincidence of 0.43%, 0.16%, and 0.07% with milking machines, feces, and bedding, respectively, with no bacteria originating from forage detected. Notably, *Pseudomonas* was identified in all three environmental samples. Other dominant genera identified included *Bacteroides imperfecta*, *Trichococcus*, *Microbacterium*, and *Flavobacterium chrysogenum* in collecting equipment; *Pseudomonas* and *Microbacterium sphaeroides* in bedding; and *Pseudomonas*, *Fusobacterium sphaeroides*, *Megalococcus*, and *Rhodobacter sphaeroides* in feces (Fig. 4B and E).

Unlike milk samples from livestock farms A and B, bedding contributed the most (0.07%) to the milk community diversity among the environmental factors considered in livestock farm C, followed by forage (0.057%), and then feces and milking machines (both at 0.04%). Furthermore, the results revealed that the raw cow’s milk from livestock farm C shared six genera (*Pseudomonas*, *Fusobacterium*, *Streptococcus*, *Enterococcus*, *Shigella*, and *Bacillus*) with bedding, two genera (*Bacillus* and *Lactobacillus*) with forage, eight genera (*Pseudomonas*, *Fusobacterium*, *Streptococcus*, *Enterococcus*, *Shigella*, *Sarcobacterium*, and *Lactobacillus*) with feces, and eight genera (*Pseudomonas*, *Fusobacterium*, *Streptococcus*, *Enterococcus*, *Shigella*, *Bacillus*, and *Corynebacterium*) with milking machines, respectively (Fig. 4C and F).

Briefly, these results illustrate that bacterial contamination of raw milk in semi-intensive and intensive livestock farms exhibits different patterns, with the former presenting a more significant pollution risk. This investigation could provide a foundation for controlling bacterial contamination in raw cow’s milk.

### Psychrophile and thermophile metabolite analysis

**Fig 5:**
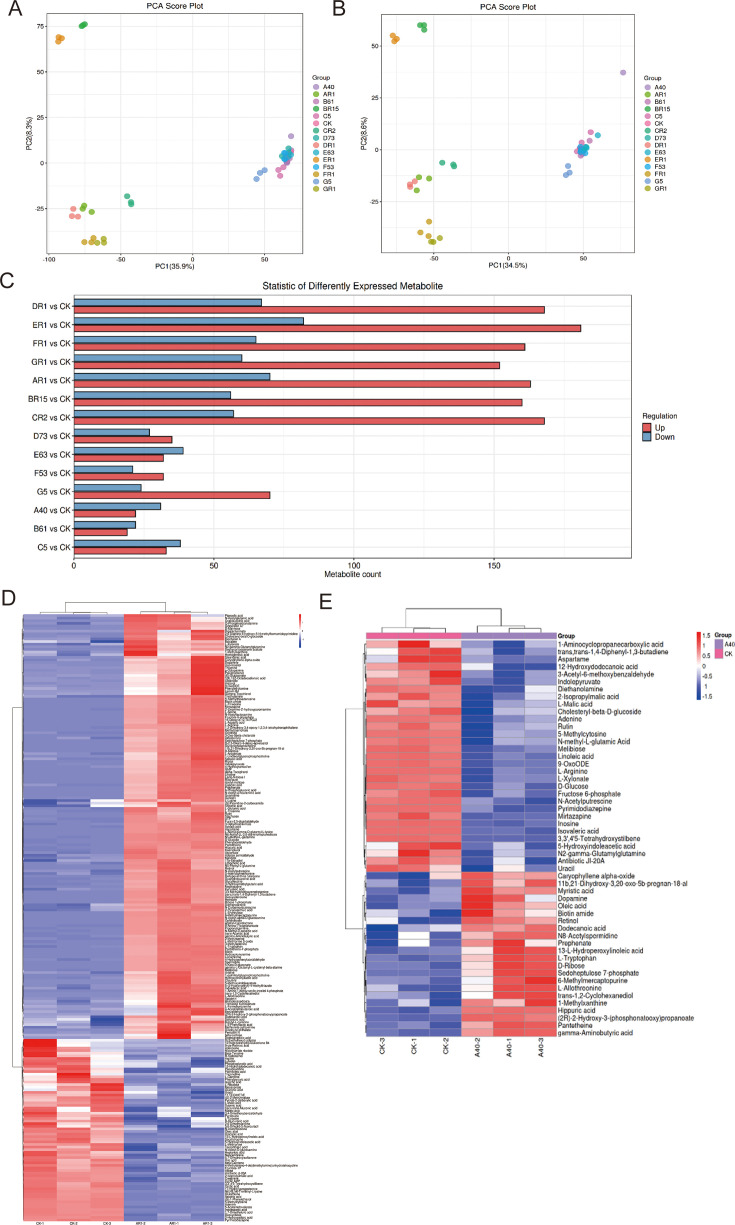
Principal component analysis (PCA) of bacterial metabolites in raw cow’s milk and heat map of differential metabolites. (**A**) Metabolite PCA plot in positive ion mode. (**B**) Metabolite PCA plot in negative ion mode. (**C**) Numbers of significantly differential metabolites detected in each strain relative to the control group. Psychrophilic strains include A40, B61, C5, D73, E63, F22, and G5, while thermophilic strains include AR1, BR2, CR2, DR1, ER1, FR1, and GR1. Red bars indicate metabolites that are significantly upregulated compared with the control (sterilized milk without inoculation), whereas blue bars indicate significantly downregulated metabolites. (**D**) Differential metabolite heatmap of *Thermus thermophilus AR1* compared with the control group (sterilized milk without inoculation). (**E**) Differential metabolite heatmap of *Thermus thermophilus A40* compared with the control group.

To further inspect the impact of psychrophiles and thermophiles on milk and derived products, as well as potential food safety concerns, their metabolites were characterized. Seven psychrophiles and seven thermophiles, which were screened from raw cow’s milk, were selected to be reintroduced into the sterilized milk, following a metabolite comparison with the no inoculation group, CK (Fig. 5).

A total of 549 metabolites were detected in positive and negative ion modes (Fig. 5A and B). PCA results demonstrated that the group inoculated with psychrophiles was more similar to the control, while the thermophiles group was significantly different from the control and produced more differential metabolites (Fig. 5C). *Bacillus licheniformis* MTE1 was the thermophile with the best capability for differential metabolites, yielding 263 species, while *Aneurinibacillus thermoaerophilus* MTG1 was the least, with 212. For psychrophiles, *Chryseobacterium timonianum* MPG5 and *Chryseobacterium timonianum* MPB61 produced 94 and 41 metabolites, respectively.

Significant up-/downregulation of several metabolites (Log_2_FC > 2/Log_2_FC < −2) was found to be prevalent in seven thermophiles (Fig. 5D). Fifty-nine metabolites were significantly up-regulated in all 7 thermophile strains, and 2 metabolites (5-methylcytosine and cytosine) were significantly downregulated. In comparison, L-tryptophan was the only significantly upregulated metabolite in all psychrophiles (Fig. 5E), and cytosine was significantly downregulated in the remaining six thermophile strains, except for *Chryseobacterium timonianum* MPG5. Besides, γ-aminobutyric acid and sedoheptulose 7-phosphate were upregulated in the four tested *Pseudomonas* strains, while four metabolites, including cytosine, honeydew, L-arginine, and N-acetyl putrescine, were significantly downregulated. Notably, 2-oxoglutarate and β-guanidinopropionic acid were upregulated in *Acinetobacter johnsonii* MPF22, N5-methylglutamine and stachyose only exhibited significant up-/downregulation in *Staphylococcus xylosus* MPE63, and the significant upregulation of ribulose-1-phosphate, 1-amino-1-deoxyinositol 4-phosphate, acetyl maltose, N6-acetyl-L-2,6-diaminoheptanedioate, inosine, galactose-1-phosphate, nicotinamide mononucleotide, and chromium luminescent and downregulation of γ-L-glutamyl-β-cysteine-L-cysteine-β-alanine were observed in *Chryseobacterium timonianum* MPG5 alone. Moreover, the upregulated metabolites of thermophilic and psychrophilic microorganisms comprised 11 toxic products in thermophiles and 1 toxic product in psychrophiles, which raised the potential risk (Table 2).

**TABLE 2:** Potential risk metabolites significantly upregulated in co-existence of thermophiles and psychrophiles

Compound name	Test organism	Administration route	LD50 (mg/kg)	Source
Pantothenylmercaptoethylamine	Mouse	Intraperitoneal	440	Thermophiles
N-Methylaspartic acid	Mouse	Intraperitoneal	137	Thermophiles
L-Alloxythreonine	Rat	Intraperitoneal	5,242	Thermophiles
Guanosine	Mouse	Intraperitoneal	500	Thermophiles
6-Methylmercaptopurine nucleoside	Mouse	Injection	115	Thermophiles
4-Acetamidobutyric acid	Mouse	Injection	425	Thermophiles
3-Methyladenine	Mouse	Intraperitoneal	280	Thermophiles
3,4-Methylenedioxyamphetamine	Mouse	Oral	13.3	Thermophiles
Adenine	Mouse	Oral	783	Psychrophiles

**Fig 6:**
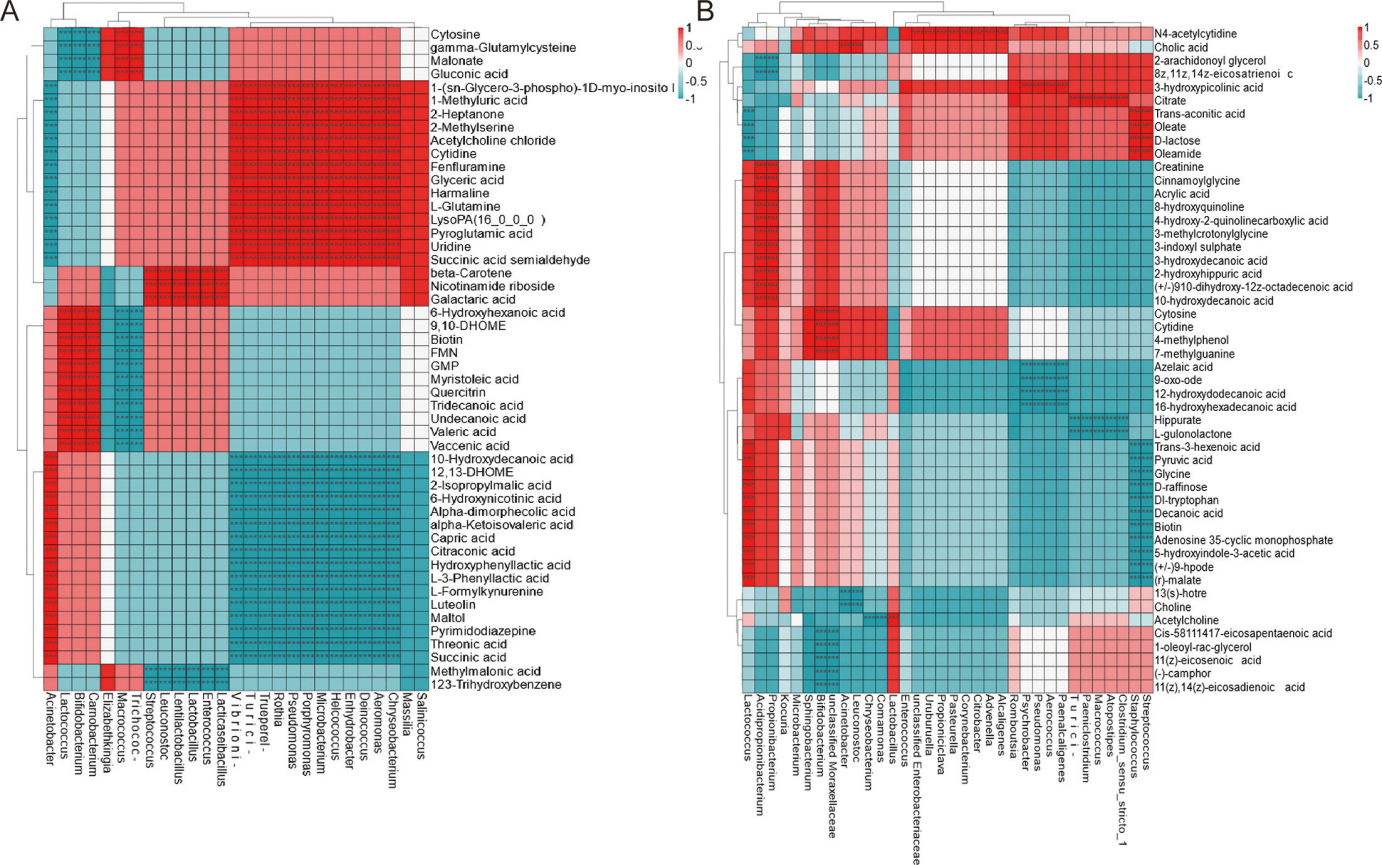
Correlation between microbial community structure and metabolites in raw milk from southern (**A**) and northern (**B**) Xinjiang livestock farms.

Setting aside the safety issue, the metabolites might also be responsible for milk’s off-tastes, as confirmed by correlation analysis (Fig. 6). Specifically, *Pseudomonas* was positively correlated with 2-heptanone (fruity and spicy) and succinic semialdehyde (irritating odor). *Acinetobacter* was associated with isobutyric acid (metamorphic flavor), succinic acid (sour), and decanoic acid (dusty flavor). *Lactococcus* influenced the presence of valeric acid (rotten flavor), pyruvic acid (sour), glycine (acetic acid flavor), L-methionine (sour, sulfur flavor), and acrylic acid (irritating odor), and *Chryseobacterium* was linked to 4-methylphenol (irritating flavor).

## DISCUSSION

The location of livestock farms plays an important role in the microbial composition of raw cow’s milk. Giannino’s study found that although the bacterial community in raw milk produced on alpine farms at different heights shared some common bacteria, the difference caused by altitude was apparent (6). Ryu et al. (25) evaluated milk in three provinces of South Korea and found a discrepancy in the core flora between rural and urban areas. Many investigations have also been conducted on the bacterial composition of raw milk. Kable et al. (26) proved that the core bacteria in raw milk from two processors in California were *Streptococcus*, *Staphylococcus*, and unidentified *Clostridium*. *Pseudomonas*, *Lactococcus,* and *Acinetobacter* are the most abundant genera found in raw milk in western German livestock farms (27). *Pseudomonas*, *Lactococcus*, *Acinetobacter*, *Enterobacter*, *Bacillus*, and *Chryseobacterium* were found to be the dominant genera in raw milk from livestock farms in different provinces of South Korea, and the proportion varied by regions (25). In our study, *Proteobacteria*, *Firmicutes*, and *Bacteroidetes* were found in the raw milk of seven livestock farms, and the former two occupied the highest proportion, which was consistent with previous studies (28, 29). At the genus level, the main bacterial flora in raw cow’s milk differs among different livestock farms and showed inconsistency with previous research. Exhaustively, *Pseudomonas* was dominant in livestock farms A, B, and E; *Lactococcus* was the main genus in C, D, and F; and *Streptococcus* was predominant in G. These results strongly supported the idea that the geographical environments shaped the microbial community and proportions of genera in raw cow’s milk, while *Lactococcus*, *Streptococcus*, *Pseudomonas*, and *Fusobacterium* generally existed. We also confirmed the influence of different livestock husbandry methods on bacterial community structure.

*Pseudomonas aeruginosa* accounted for the highest proportion of the raw cow’s milk psychrophiles in all the farms, followed by *Fusobacterium* sp. *Pseudomonas aeruginosa* accounted for 93.78% of the screened choleretic bacteria in raw milk from South Xinjiang, which indicated that *Pseudomonas aeruginosa* was the prevalent choleretic bacterium and was likely to be the dominant choleretic bacterium. Vithanage et al. and Zhang et al. (11, 30) also found that *Pseudomonas aeruginosa* was the choleretic bacterium with the highest percentage in raw milk, followed by *Bacillus fusiformis*. Meanwhile, the main *Pseudomonas* species screened at low temperatures included *Pseudomonas fluorescens*, *Pseudomonas fragilis*, *Pseudomonas ronde*, *Pseudomonas aeruginosa*, and *Pseudomonas griseus*, which have been reported (30, 31). Thermophiles, on the other hand, were mainly *Bacillus* spp. Especially, *Bacillus licheniformis* was present with a high percentage in raw cow’s milk as well as in sterilized milk and milk powder (16). Effective detection of *Pseudomonas*, *Bacillus*, and *Bacillus thiaminolyticus* should be emphasized to reduce the overall levels of psychrophiles and thermophiles in raw cow’s milk.

Milk is rich in various nutrients but also provides an ideal growth environment for microorganisms (3). Therefore, preventing milk and derivatives from contamination by microbes, including endogenous and exogenous, is a serious concern for the milk industry. Generally, exogenous pollution is the primary source of milk microbial contamination from healthy producers (32), which is attributed to post-production management conditions and environmental health problems (33). Our results exhibited that the milking machines were the most important contributor to raw milk bacterial contamination in the breeding environment, followed by litter, feces, and forage with Pseudomonas and Acinetobacter as dominant species, which was consistent with the earlier studies (34). The improper and inopportune cleaning of the milking machines, bedding, and feces might be responsible for the indirect contamination of the raw cow’s milk (1, 35). We noticed that the previously reported contamination from forage was limited in samples we explored, which could be attributed to the unique arid environment and the well management of forages (27). According to our investigation, more effort into routine cleaning of dairy farming and milking environment should be taken in semi-intensive farms, especially milk collecting equipment, feces, and bedding, which should help to lessen the contamination of *Pseudomonas* and *Fusobacterium*.

Psychrophiles can grow during low-temperature transportation of raw milk (36), and their enzymes and metabolites can affect the quality of dairy products (11). Besides, several thermophiles possess powerful resistance capabilities and can even survive thermal sterilization. For example, the spores of Bacillus can survive in heat treatment of milk, then reproduce in finished products and affect dairy products (12). Our investigation of metabolic pathways demonstrated that the galactose metabolism and arginine biosynthetic pathways in all seven strains of thermophiles significantly changed. The differential metabolites incorporated in the lactose metabolism pathway were found in all strains. Despite the quantity being between six and nine from different strains, fructose-6-phosphate, melibiose, D-glucose, and stachyose are the four common species. As for the arginine biosynthetic pathway, the number of differential metabolites was between 5 and 7, sharing four common metabolites (L-arginine, citrulline, L-aspartic acid, and L-glutamic acid). Wang et al. (37) found that the arginine biosynthesis pathway and the galactose metabolism pathway were involved in the early stage of biofilm formation, extracellular polymeric substance (EPS) production, and membrane integrity of *Bacillus licheniformis* eventually forming biofilm. Our results suggest that thermophiles may contribute to biofilm-related processes, such as extracellular polymeric substance production, as reported in *Bacillus licheniformis* (37). Although we did not directly quantify biofilms, these metabolic signatures indicate potential biofilm involvement. The observation of upregulation of γ-amino acids and downregulation of L-arginine and N-acetylputrescine in the three mentioned strains was consistent with the L-arginine decomposition pathway of *Pseudomonas putida* studied by Miller and Rodwell (38). 4-Acetylaminobutyric acid, L-allothreonine, L-serine, L-methionine sulfoxide, and L-tryptophan are the products involved in amino acid metabolisms of psychrophiles/thermophiles, probably alluding to their different metabolic pathways for protein degradation. Wolfe et al. (39) showed that the valine, leucine, isoleucine, and other amino acid degradation pathways were related to irritating odors such as volatile sulfides. Free amino acids will first increase the metabolism of cysteine and methionine under the action of bacteria and then continue to form a variety of biogenic amines through bacterial metabolism, which will further lead to food corruption and toxicity (40, 41). Compounds such as 3,4-methylenedioxyamphetamine may originate from environmental or feed sources rather than microbial biosynthesis alone. Although not routinely monitored in dairy, such compounds raise food safety concerns. According to the EU Commission Regulation No. 1881/2006 and Chinese GB 2762-2022, maximum residue limits for psychotropic substances in milk are not established, highlighting a monitoring gap. We strongly recommend detecting and controlling psychrophiles and thermophiles during the storage and transportation of dairy products, minimizing the impact of their metabolites, and improving milk and derivative product quality.

The number of replicates per farm was principally constrained by COVID-19 control measures in 2023, which restricted entry of external researchers into production areas and necessitated reliance on trained farm staff for specimen collection. Although this limitation reduced the number of replicates, our sampling scheme intentionally included geographically distant and representative sites—three Nanda farms plus one Xiyuchun farm in Kashgar and three Xiyuchun farms in Changji—all of which are large, nationally standardized operations. This design helped ensure that the most representative herds were captured despite pandemic-related restrictions. Nevertheless, the limited replicate number remains a study limitation; future work with expanded temporal, seasonal, and increased sample sizes will be required to validate and extend these findings.

### Conclusion

In this study, key psychrophiles and thermophiles in raw cow’s milk were identified through a combination of traditional culture and high-throughput sequencing, their potential hazards to the quality and safety of raw cow’s milk and dairy products were investigated through enzyme activity characterization and metabolomics, and principal contamination sources were determined. Our results have been communicated to the cow milk production company to facilitate reducing contamination and introduced risks. Based on our results and previous reports, the key psychrophiles and thermophiles and their common differential metabolites are expected to serve as risk markers for raw milk production, storage, and transportation for dairy enterprises in Xinjiang, which could be further used for rapid detection and monitoring of psychrophiles and thermophiles and expand their application in the dairy industry. Besides, the annotation results showed several strains possess low identity with the records in the NCBI database, which suggests potential novel strains.

## Data Availability

The 16S rRNA sequencing data generated in this study have been deposited in the NCBI Sequence Read Archive under BioProject accession number PRJNA1322133. The metabolomics data set has been deposited in the Metabolomics Workbench under Study ID ST004319 (https://www.metabolomicsworkbench.org).

## SUPPLEMENTAL MATERIAL

Supplemental material aem.01065-25-s0001.pdf High-resolution version of Fig. 5D.

ASM does not own the copyrights to Supplemental Material that may be linked to, or accessed through, an article. The authors have granted ASM a non-exclusive, world-wide license to publish the Supplemental Material files. Please contact the corresponding author directly for reuse.
