# Supplementary figures and images for: Community structure and metabolite profiles of psychrophiles and thermophiles in bovine raw milk from arid inland areas
Source: Appl Environ Microbiol. 2026 May 15;92(6):e01065-25. doi: 10.1128/aem.01065-25 (PMC13274413; doi:10.1128/aem.01065-25)

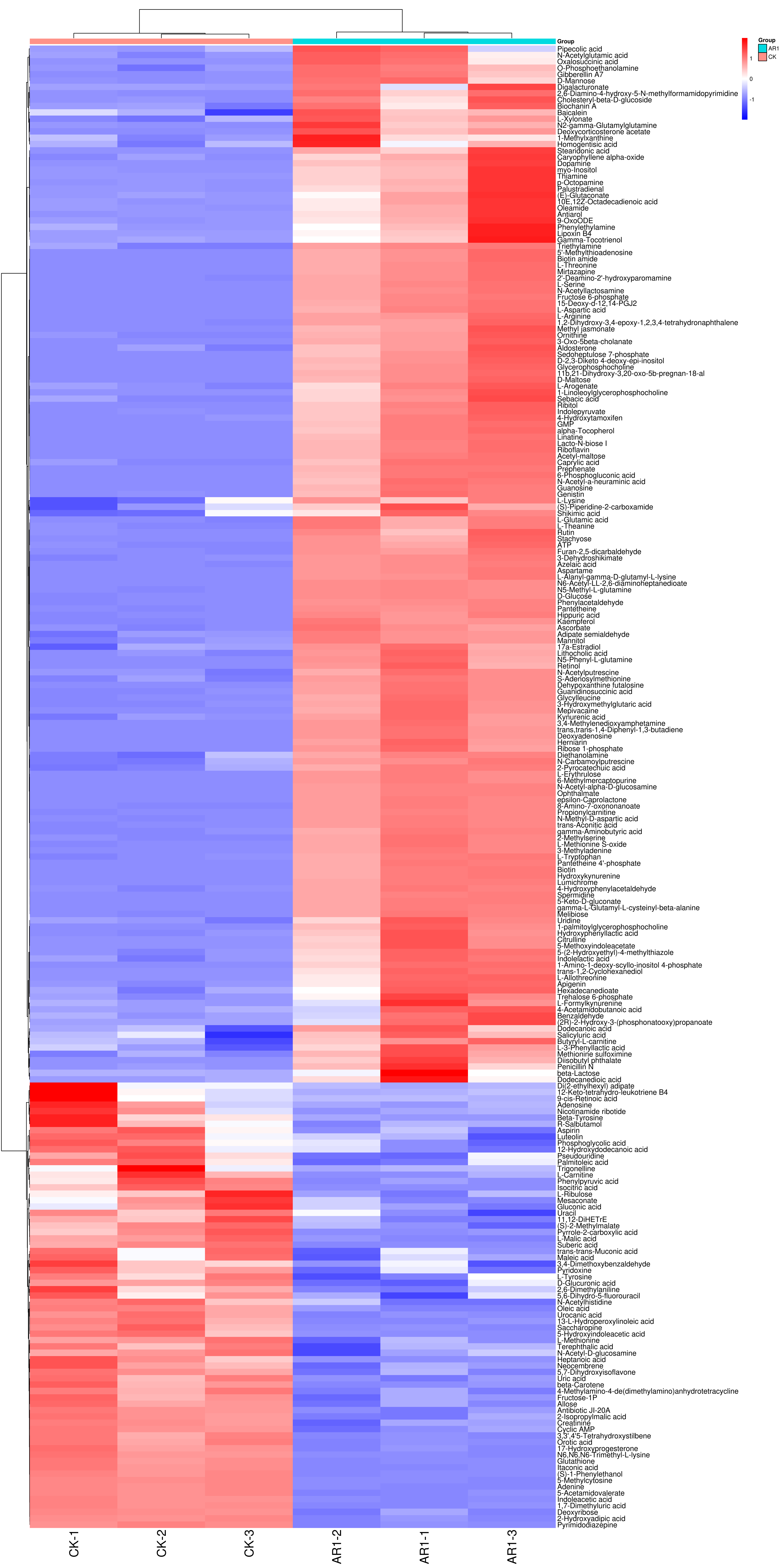

Supplement: Supplemental material — High-resolution version of Fig. 5D. [file aem.01065-25-s0001.pdf]
